# Effect of Age and Weaning on Growth Performance, Rumen Fermentation, and Serum Parameters in Lambs Fed Starter with Limited Ewe–Lamb Interaction

**DOI:** 10.3390/ani9100825

**Published:** 2019-10-18

**Authors:** Shiqin Wang, Tao Ma, Guohong Zhao, Naifeng Zhang, Yan Tu, Fadi Li, Kai Cui, Yanliang Bi, Hongbiao Ding, Qiyu Diao

**Affiliations:** 1 State Key Laboratory of Grassland Agro-Ecosystems, Key Laboratory of Grassland Livestock Industry Innovation, Ministry of Agriculture and Rural Affairs, College of Pastoral Agriculture Science and Technology, Lanzhou University, Lanzhou 730020, China; wshq1988@163.com (S.W.); lifd@lzu.edu.cn (F.L.); 2 Feed Research Institute, Chinese Academy of Agricultural Sciences/Key Laboratory of Feed Biotechnology of the Ministry of Agriculture and Rural Affairs, Beijing 100081, China; matao@caas.cn (T.M.); zhaoguoh@foxmail.com (G.Z.); zhangnaifeng@caas.cn (N.Z.); tuyan@caas.cn (Y.T.); cuikai@caas.cn (K.C.); vetbi2008@163.com (Y.B.); dinghongbiao@caas.cn (H.D.)

**Keywords:** early weaning, weaning stress, lamb, growth performance, diarrhea

## Abstract

**Simple Summary:**

Early weaning is a common practice in the modern lamb industry, which shortens the breeding cycle of ewes and improves the flock productivity by increasing the frequency of lambing. Recently, it was suggested that weaning age (21 or 35 days of age) and milk replacer feeding level had limited effect on apparent digestibility of nutrients in lambs. However, artificial rearing may increase the cost due to the use of milk replacer, labor, and feeding facilities. In this study, we compared the difference in growth performance of lambs weaned at 21 days (early weaning) and 49 days (conventional weaning) of age. All lambs were initially reared with ewes and supplemented with starter at 7 days of age. The results showed that diarrhea rate was increased when lambs were weaned at 21 days of age while average daily gain was decreased. Early weaning reduced average daily gain without compromising lambs’ overall immunity. In conclusion, early weaning (21 days of age) may have a negative impact on lambs’ performance based on a short-term study.

**Abstract:**

Sixty neonatal Hu lambs were weaned at either 21 (n = 30) (early weaning, EW) or 49 days (n = 30) of age (control, CON). The starter intake and body weight (BW) of lambs was recorded weekly from birth to 63 days of age. Diarrhea rate of lambs was measured from birth to 35 days. Six randomly selected lambs from each treatment were slaughtered at 26, 35, and 63 days of age, respectively. Ruminal pH, NH_3_-N, and volatile fatty acid (VFA) concentration, as well as serum parameters including immunity, antioxidant status, and inflammatory parameters from randomly selected lambs from each treatment were measured. There was no difference in BW at birth and day 21 between the two groups of lambs (*p* > 0.05). However, BW of the lambs in the EW group was significantly lower than those in the CON group (*p* < 0.01) from 28 to 49 days of age. Average daily gain (ADG) of the lambs in the EW group was significantly lower than those in the CON group (*p* < 0.01) at three weeks after early weaning. Starter intake of the lambs in the EW group was obviously higher than that in the CON group (*p* < 0.01) from day 28 to 49. In addition, the diarrhea rate was significantly higher than that in the CON group from day 5 to 14 after weaning (*p* < 0.01). The EW group had heavier carcasses (*p* < 0.01) and rumen relative to whole stomach weights (*p* < 0.01). Rumen pH was increased by age (*p* < 0.01) and was not affected by early weaning (*p* > 0.05). Early weaning decreased abomasum relative to whole stomach weight (*p* < 0.01) and increased total VFA concentrations (*p* < 0.01) at day 26. There was no difference in lambs’ immunity and stress indicators (*p* > 0.05). The results indicated that lambs weaned at 21 days of age had decreased ADG and higher diarrhea rate, although the overall immunity was not compromised. Long-term study is needed to further validate the feasibility of early weaning strategy in lambs.

## 1. Introduction

Weaning time is critical to the management of ewes and lambs. Although there is no ‘best’ age to wean, lambs can be weaned at as early as three-weeks old [1], and early weaning is a common practice in modern lamb industry. Early weaning shortens the breeding cycle of ewes, which improves the flock productivity by increasing the frequency of lambing [2,3]. More importantly, lambs utilize diet more efficiently than ewes because lambs directly convert feed to gain while ewes convert the feed to milk and then to lamb gain. Therefore, an increase in economic profits can be expected if early weaning is successful. However, early weaning can be an important stressor for lambs due to separation from dams [4], as it is suggested that suckling is a major factor in the strength of the ewe–lamb contact [5]. In addition, the transition from liquid (milk/milk replacer) to solid diets (starter or grass) during weaning requires the development of a functional rumen [6]. Consequently, early weaning may cause a drop in feed consumption and decrease in growth rate of lambs [7] if the rumen structure and function has not been fully developed before weaning [8].

To minimize the negative effect of early weaning as well as ensure economic benefits, great efforts have been made to investigate the optimal weaning strategy for lambs. Early study suggested that lambs reared under a mixed system, featured by separating lambs from their dams for 15 h while allowing them to suckle for the remaining 9 h daily, had better financial return than those exclusively suckling their dams and weaned at 30 days after birth [9]. On the other hand, it is reported that sufficient starter intake can stimulate the development of a functional rumen as well as the establishment of rumen microbiota [6]. Our previous study suggested that when starter was offered at day 15 of age, lambs separated from their dams at 20 days of age and artificially fed milk replacer had superior growth performance than their ewe-reared counterparts at the end of the feeding trial (90 days of age) [10]. Recently, it was suggested that weaning age (21 or 35 days of age) and milk replacer feeding level had limited effect on apparent digestibility of nutrients, ruminal microbiota, and fermentation of lambs at 50 days [11]. However, artificial rearing may increase the cost due to the involvement of milk replacer, labor, and feeding facilities [12].

Here, we compared the effect of limited ewe–lamb contact (18 h daily) and supplementation of starter at an early age (seven-days old) on growth performance, rumen fermentation, slaughter performance, and serum parameters of lambs weaned at either 21 or 49 days of age in a 63-day feeding trial. We hypothesized that early weaning (21 days of age) may have temporal negative effect on lambs’ performance, but the overall performance of lambs may be similar between two groups.

## 2. Materials and Methods

The study was conducted from October to December, 2018 at Runlin sheep farm (N 36°82′, E 115°83′) in Linqing county, Liaocheng city, Shandong province, China. The experiment protocol was approved by the Animal Ethics and Humane Animal Care of the Chinese Academy of Agricultural Sciences (with protocol FRI-CAAS-20180810).

A total of 60 neonatal Hu lambs (48 male and 12 female) with similar birth weights (3.82 ± 0.46 kg) were used. Lambs were initially reared with ewes in 6 well-ventilated sheep pens (4 m × 5 m) with controlled temperature and humidity, with 10 lambs (8 male and 2 female) and their ewes in each pen. All ewes were fed two times daily according to the farm’s feeding management schedule. None of the lambs in the groups had access to the ewes’ feed. Ewes were fed a total mixed ration consisting of 35% corn silage, 28% peanut straw, 7% garlic straw, 3% soybean residue, 15% corn, 6% wheat bran, and 6% soybean meal. 

From day 7 of age, all lambs were separated from the ewes and offered pelleted starter for 6 h daily (08:00–10:00, 12:00–14:00, and 16:00–18:00). At other times, the lambs remained with their dams and still had free access to starter. All lambs were fed starter 1 from 7 to 35 and starter 2 from 36 to 63 days of age, respectively. The ingredient and nutritional composition of starters are listed in Table 1. Half of the lambs from three pens (n = 30) were weaned and abruptly separated from their dams at 21 days of age (early weaning, EW) while the other half of the lambs from another three pens (n = 30) were weaned and abruptly separated from their dams at 49 days of age (control, CON). All lambs and ewes had ad libitum access to water. During the experiment, two lambs from the CON group did not have enough milk intake as their dams were sick, and one lamb from the CON group died accidentally. Therefore, those three lambs were removed from further analysis.

The body weight (BW) of each lamb was recorded weekly (before morning feeding) from 7 to 63 days of age. The starter was offered to each pen and the intake of starter was measured by each pen daily before morning feeding. Representative samples of starter were collected weekly and were immediately frozen at −20 °C for further analysis. Diarrhea incidence was also monitored on a daily basis from birth to 35 days of age.

Blood sampled from six male lambs per treatment was collected in heparinized tubes by jugular venipuncture before morning feeding at 26, 35m and 63 days of age, respectively. The blood sample was centrifuged at 3500× *g* for 15 min at 4 °C to separate serum, which was pipetted into 2 mL cryotubes and stored at −20 °C until subsequent analysis. After blood sampling, those six lambs from each group were slaughtered at 26, 35, and 63 days of age, respectively. Ruminal digesta sample was collected and pH value was measured immediately. The rumen digesta was then filtered through four layers of cheesecloth and a 10 mL subsample of each strained fluid was collected, stored at −20 °C for analysis of the volatile fatty acids (VFAs) and ammonia nitrogen (NH_3_-N). Immediately after slaughter, the rumen, reticulum, omasum, and abomasum of each lamb was dissected and weighted after the digesta was removed. The carcass weight of each lamb was also recorded.

The starter samples were ground to pass through a 1 mm sieve and dried in an oven at 135 °C for 2 h (method 930.15; AOAC, 1990) [14] to measure the dry matter (DM) content. The ash content, nitrogen, neutral detergent fiber (NDF), acid detergent fiber (ADF), calcium, and total phosphorus was measured according to methods described by previous studies [14,15,16,17]. Crude protein (CP) was calculated as 6.25 × Nitrogen. 

Feces were scored using a 1 to 4 scale classified as firm and well-formed (score 1), soft and pudding-like (score 2), runny and pancake batter-like (score 3), or liquid and splatters (score 4) as described previously [18]. If an animal presented a fecal score ≥ 3 for 3 consecutive days, it was considered diarrheic. Diarrhea rate (%) = number of lambs of diagnosed at least once for diarrhea/the total number of lambs. Diarrhea frequency (%) = the number of diarrhea lambs × diarrhea days/the total number of lambs × experimental days.

Serum concentration of immunoglobulin (IgG, IgM, and IgA), superoxide dismutase (SOD), Glutathione Peroxidase (GSH-Px), catalase (CAT), total antioxidative capacity (T-AOC), and malondialdehyde (MDA) were determined using the Hitachi 7020 autobiochemistry instrument (Hitachi, Tokyo, Japan) with corresponding commercial test kits (Nanjing Jiancheng Bioengineering Institute, Nanjing, Jiangsu, China) following the manufacturer’s instructions. Interleukin 1β (IL-1β), interleukin 4 (IL-4), interleukin 6 (IL-6), cortisol, interferon-gamma (IFN-γ), and tumor necrosis factor α (TNF-α) were determined using bovine ELISA kits (Nanjing Jiancheng Bioengineering Institute, Nanjing, Jiangsu, China) following the manufacturer’s instructions.

Individual and Total VFA concentrations were determined as described previously [19]. Briefly, 1 mL rumen fluid filtrate was added to 0.2 mL of metaphosphoric acid solution (250 g/L) containing 2 g/L 2-ethyl butyrate, mixed overnight, and analyzed by gas chromatography (SP-3420, Beijing Analytical Instrument Factory, Beijing, China). The concentration of NH_3_-N was measured using phenol hypochlorite colorimetric method as described by [20] 

Starter intake, BW, and average daily gain (ADG) of lambs from each group were pooled by week. Data for weights of carcasses and stomach compartment, blood variables, as well as rumen fermentation parameters of lambs from each group were pooled at day 21, 35, and 63, respectively. Those data were analyzed according to a two-way ANOVA using PROC GLM of SAS (SAS Inst. Inc., Cary, NC, USA) to examine the effect of treatment, age, and the interaction between treatment and age. The statistical model was as follows:

Yij = μ + Ti + Aj + TAij + Eij(1)

where Yij is the dependent variable, μ is the overall mean, Ti is the treatment effect, Aj is the age effect, TAij is the interaction of treatment and age, and Eij is the error term.

One-way ANOVA was used to analyze the effect of weaning time on diarrhea rate and diarrhea frequency. The statistical model was as follows:

Yi = μ + Ti + Ei(2)

where Yi is the dependent variable, μ is the overall mean, Ti is the Treatment effect, and Ei is the error term. Multiple comparisons of means among treatments were performed using Tukey’s tests. Significance was defined as *p* < 0.05.

## 3. Results

### 3.1. Growth Performance and Diarrhea Rate

**Figure 1:**
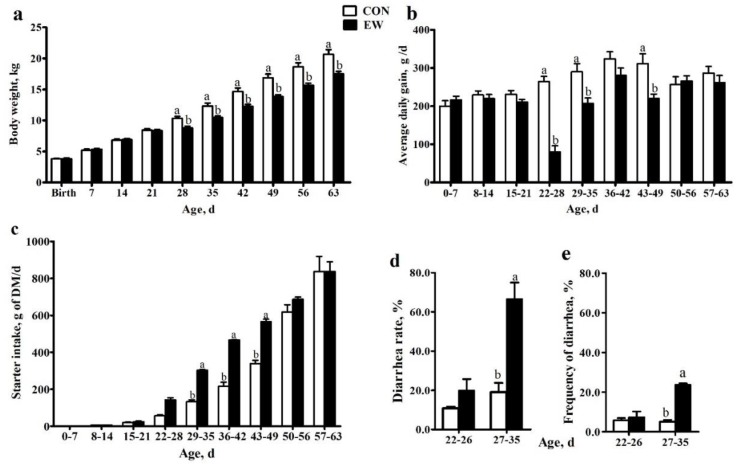
Body weight (**a**), average daily gain (**b**), starter intake (**c**), and diarrhea rate (**d**,**e**) for lambs weaned at day 21 as early weaned group (EW; black columns) or lambs weaned at day 49 as conventional weaned group (CON; white columns). Columns with different letters at a single time point indicate means that differed based on the means separation (*p* < 0.05). Error bars indicate SEM for the treatment × age interaction.

There was no difference in BW from birth until day 21 of age between the two groups of lambs (*p* > 0.05) (Figure 1a). However, BW of lambs in the EW group was significantly lower than BW in the CON group (*p* < 0.05) after 21 days of age (Figure 1a). No difference was observed in ADG at 21 days of age between two groups of lambs (*p* > 0.05) (Figure 1b). The ADG of lambs in the EW group was significantly lower than ADG of those in the CON group at 28 (*p* < 0.01), 35 (*p* < 0.001), 42 (*p* < 0.001), and 49 (*p* < 0.001) days of age (Figure 1b). 

No difference was observed in starter intake from 8 to 28 days of age between the two groups of lambs (*p* > 0.05) (Figure 1c). The starter intake of lambs in EW group was significantly higher than that in CON group (*p* < 0.05) from 29 to 49 days of age (Figure 1c). No difference in starter intake between two groups of lambs was observed from 50 to 56 (*p* > 0.05) and 57 to 63 days of age (*p* > 0.05) (Figure 1c). 

No diarrhea incidence was observed for any lamb from birth to 21 days of age. From 22 to 26 days of age, the diarrhea rate as well as frequency was not different between lambs in EW and CON group (*p* > 0.05) (Figure 1d). From day 27 to 35, the diarrhea rate and frequency of lambs in EW group was significantly higher than that in CON group (*p* < 0.001). No diarrhea incidence was observed after 35 days of age for lambs in both groups.

### 3.2. Slaughter Performance and Development of Visceral Organs

Both slaughter BW (*p* = 0.010) and hot carcass weight (*p* = 0.007) of lambs from EW group was significantly lower than that of lambs from CON group (Table 2). Dressing percentage decreased over time for both groups (*p* < 0.001) and tended to be lower in EW than CON group (*p* = 0.065). No change was found in the weight of whole stomach (*p* = 0.705), but abomasum relative to whole stomach weight (*p* = 0.003) was lower, while rumen (*p* = 0.007) and omasum (*p* = 0.035) relative to whole stomach weight was higher for lambs in EW than those in CON group. Reticulum relative to whole stomach weight was not different between two groups of lambs (*p* = 0.821). Both treatment and age interactively affected dressing percentage (*p* = 0.021) and reticulum relative to whole stomach weight (*p* = 0.049).

### 3.3. Rumen Fermentation Parameters

Both rumen pH (*p* = 0.007) and NH_3_-N concentration (*p* < 0.001) increased over time and was not affected by treatment (*p* = 0.694 and 0.999, respectively) (Table 3). Total VFA concentration was higher in EW than CON group (*p* = 0.027). Molar proportion of acetate (*p* = 0.804), propionate (*p* = 0.154), butyrate (*p* = 0.171), isobutyrate (*p* = 0.991), isovalerate (*p* = 0.159), or acetate/propionate ratio (*p* = 0.253) was not affected by treatment. Molar proportion of valerate tended to be higher in the EW than that in the CON group (*p* = 0.098). Treatment and age interactively affected the NH_3_-N concentration (*p* = 0.040) and molar proportion of isovalerate (*p* = 0.041).

### 3.4. Serum Parameters

The concentration of IgG (*p* = 0.761), IgA (*p* = 0.809) or IgM (*p* = 0.889) was not affected by treatment (Table 4). The concentration of IgG (*p* < 0.001) was lower, while that of IgA (*p* < 0.001) and IgM (*p* < 0.001) was higher at 63 than 26 days of age for both groups. No difference was observed in activity of SOD (*p* = 0.839), GSH-Px (*p* = 0.290), CAT (*p* = 0.145), T-AOC (*p* = 0.605), or MDA (*p* = 0.464) between the two groups of lambs (Table 4). Activity of SOD (*p* < 0.001), GSH-Px (*p* < 0.001), CAT (*p* < 0.001), and T-AOC (*p* < 0.001) was higher, while that of MDA (*p* < 0.001) was lower at 35 than those at 26 or 63 days of age for both groups. There was no difference in the concentration of IL-1β (*p* = 0.659), IL-4 (*p* = 0.361), IL-6 (*p* = 0.085), cortisol (*p* = 0.946), IFN-γ (*p* = 0.843), or TNF-α (*p* = 0.927) between the two groups of lambs. The concentration of IL-1β (*p* < 0.001), IL-4 (*p* < 0.001), IL-6 (*p* < 0.001), cortisol (*p* < 0.001), IFN-γ (*p* < 0.001), and TNF-α (*p* < 0.001) were higher, while that of MDA (*p* < 0.001) was lower at 35 than those in 26 or 63 days of age for both groups. There was no interactive effect of treatment and age on any serum parameter (*p* > 0.05).

## 4. Discussion 

It has been generally accepted that intake of solid feed before weaning stimulates rumen development and functionality [21,22]. Early starter feeding (7 days of age) was reported to increase ruminal VFA concentration in lambs compared with late starter feeding (42 days of age) [23]. Therefore, lambs were provided starter at 7 days of age in the current study. However, a sharp decrease in ADG (from 21 to 28 days of age) of lambs weaned at 21 days of age was not prevented. This might be partly due to the short time for ewe-lamb separation (6 h daily), which was insufficient for lambs to get used to the abrupt separation from their dams at 21 days of age. The final BW of lambs weaned at 21 days of age was still lower than that of their counterparts weaned at 49 days of age, which again proved that early-life stress may have a long-lasting negative effect on the performance of lambs. For example, it is suggested that lambs subjected to nutrient restriction at 15 days of age followed by realimentation at 60 days of age still physically lag behind those continuously provided sufficient nutrients, featured by a lower BW at 90 days of age (24.1 vs. 26.1 kg) [24]. On the other hand, we found that the growth performance of lambs weaned and separated from their dams at 49 days of age (>20 kg of BW at 60 days of age) was better than those weaned and separated from their dams at 60 days of age (12.8 kg of BW at 60 days of age) [25]. This discrepancy might be explained by the difference in time of introduction of starter to lambs between current (day 7) and our previous study (day 15), as well as the higher protein and energy level of starter used in the current study than our previous study [25]. 

Post-weaning diarrhea is an economically important enteric disease due to financial losses [26]. In our present study, diarrhea rate and frequency were increased in the EW group due to early weaning. Similarly, this disease occurs most frequently within the two weeks after weaning and is characterized by a profuse diarrhea, dehydration, significant mortality, and loss of body weight of pigs [27]. Our previous study showed a sudden change in the feeding system due to early weaning resulted in changes of the intestinal morphology and functions [28], that may trigger reductions in digestive and absorptive capacities. Studies suggest that increasing weaning age reduces stress associated with this period and allows animals to have a more mature gastrointestinal tract and become increasingly familiar with solid feed during milk-period with an improvement in growth performance [29,30].

Ruminal pH is crucial for normal development, fermentation, and overall lamb health. Generally, rumen fluid pH is influenced by the rate of fermentation and absorption of VFA, which in turn, are affected by passage rate of digesta and the buffering capacity of the rumen contents [31]. In this experiment, rumen pH of lambs on day 26 or day 35 were lower than day 63 and not affected by weaning time, mainly due to the difference in starter intake between pre-and post-weaning. The increase in pH with age may be explained by the continuous development of rumen and starter intake. Furthermore, when the rumen epithelium is still underdeveloped, early starter feeding in lambs can increase ruminal VFA concentrations and affect the expression levels of genes involved in VFA transport and pH regulation in the ruminal epithelium [23]. 

Previous studies showed that ruminal VFA concentration increased with increasing solid feed intake and was affected by either dietary (e.g., nutrient fractionation) or animal factors (e.g., absorption and passage rate) [6]. Rumen development is not only driven by the presence of VFA, but also by the nutrients supplied from liquid feed [6]. In our study, weaning and feeding starter to lambs at day 21 promoted the development of the rumen, which is in accordance with the results in previous studies as providing solid feed as early as possible is beneficial to the rapid development of the rumen [32,33]. In the current study, the concentration of Total VFA, molar proportion of acetate and propionate in the EW group increased only at 5 days after weaning. This is because more starter intake in the EW group promoted rumen development, while the CON lambs were mainly fed milk. The consumption of starter in lambs has been shown to increase the ruminal concentrations of VFAs, which have positive effects on the development of ruminal epithelium [23]. This result is similar with Yang et al. [34] who showed no difference in Total VFA concentrations in lambs between pre- and post-weaning.

The effect of passive immunity on mortality, morbidity, and subsequent growth of newborn animals is receiving increasing attention [35]. Among three major immunoglobulin isotypes, namely IgG, IgM, and IgA, IgG usually has the highest concentration in serum and is the main antibody for humoral immunity. A previous study showed that serum IgG concentration was positively associated with calf health, as the morbidity and intensity of disease were the lowest in heifer calves with serum IgG concentration exceeding 10 g/L at 30 to 60 h of life [36]. In our study, the IgG concentration was higher than that of lambs (same age) reported by Hernández-Castellano et al. [37], which may due to the difference in sheep species or different test method of immunodiffusion, as a recent review suggested that the concentration of IgG may differ between methods such as ELISA or radial immunodiffusion [35]. In the present study, no change was found in the concentration of IgG, IgA, or IgM. Similarly, our previous study also showed no difference in serum IgG and IgA between lambs differing in weaning time [25]. Those results are in agreement with Hernández-Castellano et al. [37] who found no difference in the IgG concentration pre- and post-weaning, which may be due to the beginning of the endogenous immunoglobulin production by lambs and increase in the concentration of IgG over time. The authors also found the concentrations of IgG and IgM in blood of lambs decreased after birth with aging until the end of milk feeding period and then increased 30 days after weaning, and this phenomenon is not affected by milk sources (nursing, milk replacer, or cow milk) [37]. In our study, during the first few days of birth, serum immunoglobulin may typically reflect the efficacy of passive immunity transfer through adequate and timely consumption of colostrum. Later on, as feed intake increased, the systematic humoral immune function of lambs developed.

GSH-Px is an antioxidant enzyme that helps to control the levels of hydrogen peroxide and lipid peroxides under normal metabolic conditions. T-AOC indicates the oxidation resistance capacity of the whole body. MDA is one of the final products of polyunsaturated fatty acid peroxidation in cells and is considered as a marker of oxidative stress. The insignificant difference of GSH-Px, T-AOC, and MDA between lambs in different treatments suggested that the antioxidant status was not affected by weaning time. The activity of SOD, GSH-Px, CAT, and T-AOC was the highest, while that of MDA concentration was the lowest at 35 days of age for both groups, suggesting that antioxidant status improved with age. It was reported that weaning induced lower activities of antioxidant enzymes and a greater content of MDA, reflecting an imbalance of redox status in piglets [38]. However, Buchet et al. [39] reported that the observed effects are related with weaning but not with age.

In our study, no changes in the concentrations of cortisol were observed in treatment. Previous study suggested that the plasma concentration of cortisol increased in calves when they were subjected to weaning [40]. On the contrary, it was reported that the change in plasma cortisol concentration was not affected by weaning [41], and a similar result was observed in lambs [42] and piglets [43]. This may be related to the sampling time after weaning, because inflammatory response is a short-term change that only occurs within a few hours after weaning and does not last for a long time [40,44].

Several cytokines biomarkers such as IL-1β, IFN-γ, and TNF-α were used to evaluate stress and inflammatory response before and after weaning [40,42]. Early weaning can stimulate the secretion of proinflammatory cytokines such as IL-1, TNF-α, and stimulate inflammatory response [42]. Yang et al. [45] found that plasma IFN-γ declined with age during weaning period in calves. TNF-α often work synergistically with IFN-γ and IL-1β to improve immune responses [46]. Study in lambs have shown that serum TNF-α concentration was not affected by early weaning or age [25,42], and are responsive to proinflammatory cytokine regulation due to early weaning. In our study, no changes in the concentrations of IL-1β, IL-4, IL-6, IFN-γ, or TNF-α were observed in response to weaning time. Therefore, more sampling time is needed in future study to validate the findings in the current experiment.

## 5. Conclusions

The results indicate that early starter feeding combined with limited ewe-lamb contact promoted rumen development and increased Total VFA concentration, without compromising lambs’ overall immunity weaning at 21 days of age. However, the increase in diarrhea rate and decrease in ADG in lambs weaned at 21 days of age suggested that a long-term effect of weaning on overall performance as well as development in gut microbiota and barrier function needs to be assessed.

## Figures and Tables

**Table 1:** Ingredients and chemical composition of the starters.

Item	Starter 1 (7–35 Days)	Starter 2 (36–63 Days)
Ingredients, air dry basis, %		
Alfalfa hay		7.0
Oat grass		5.0
Corn	50.0	45.5
Soybean meal	23.5	20.0
Corn germ meal	12.0	10.0
Wheat bran	10.0	8.0
Vitamin and mineral mixture ^1^	1.0	1.0
Limestone	2.0	2.0
CaHPO_4_	0.5	0.5
Salt	0.5	0.5
NaHCO_3_	0.5	0.5
Chemical composition ^2^, % of DM		
DM, air dry basis	90.2	89.1
CP	21.5	21.5
NDF	15.1	18.9
ADF	6.4	8.2
Ash	8.0	5.7
Ca	0.9	0.8
P	0.5	0.6
ME, MJ/kg	10.9	10.5

^1^ Contained per kilogram of supplement: 800,000 IU of vitamin A, 300,000 IU of vitamin D3, 3000 mg of vitamin E, 4 g of Fe, 4 g of Mn, 0.8 g of Cu, 5 g of Zn, 20 mg of Se, 70 mg of I, 40 mg of Co. ^2^ DM, CP, NDF, Cam and P were measured values, while ME (metabolizable energy) was calculated according to National Research Council 2007 [13].

**Table 2:** Carcass weight, weight of rumen, reticulum, omasum, and abomasum relative to whole stomach weight on 26, 35, and 63 days of age ^1^.

Item	Treatment	Day of Age			SEM ^2^	*p*-Value ^2^		
		26	35	63		Treatment	Age	Treatment × Age
Slaughter BW, kg	CON	9.8 ^bc^	12.3 ^b^	22.2 ^a^	0.779	0.010	<0.001	0.675
	EW	8.8 ^c^	10.2 ^bc^	20.0 ^a^				
Hot carcass, kg	CON	4.7 ^b^	5.9 ^b^	10.2 ^a^	0.443	0.007	<0.001	0.376
	EW	4.4 ^b^	4.5 ^b^	8.7 ^a^				
Dressing percentage, %	CON	48.3 ^ab^	47.6 ^abc^	45.8 ^bdc^	0.491	0.065	<0.001	0.021
	EW	49.8 ^a^	44.4 ^dc^	43.5 ^d^				
Whole stomach, g	CON	135.5 ^c^	237.0 ^b^	566.6 ^a^	21.400	0.705	<0.001	0.936
	EW	127.7 ^c^	238.7 ^b^	552.7 ^a^				
relative to whole stomach weight, %								
Rumen	CON	45.1 ^e^	55.5 ^dc^	67.9 ^ab^	1.784	0.007	<0.001	0.300
	EW	51.3 ^de^	61.0 ^bc^	68.9 ^a^				
Reticulum	CON	7.8	9.1	9.9	0.502	0.821	0.258	0.049
	EW	9.1	8.7	8.7				
Omasum	CON	4.5 ^bdc^	3.7 ^d^	6.3 ^ab^	0.434	0.035	<0.001	0.648
	EW	5.7 ^abc^	4.1 ^dc^	7.0 ^a^				
Abomasum	CON	42.6 ^a^	31.7 ^b^	15.9 ^c^	1.852	0.003	<0.001	0.102
	EW	33.9 ^b^	26.2 ^b^	15.4 ^c^				

^1^ Lambs weaned at day 21 as early weaned group (EW) or lambs weaned at day 49 as conventional weaned group (CON). Values are presented as means; n = 6 per group, BW= body weight. ^2^ Treatment = the effect of weaning time; age = the effect of age; treatment × age= the interaction between treatment and age. SEM = standard error of the mean. ^a–^^d^ Means within two rows (six values including age and treatment) with different superscripts differ (*p* < 0.05).

**Table 3:** Rumen pH, ammonia nitrogen (NH_3_-N), and volatile fatty acid (VFA) profiles of lambs at 26, 35, and 63 days of age ^1^.

Item	Treatment	Day of Age			SEM ^2^	*p*-Value ^2^		
		26	35	63		Treatment	Age	Treatment × Age
pH	CON	5.7 ^b^	5.5 ^b^	6.3 ^a^	0.081	0.694	0.007	0.684
	EW	5.6 ^b^	5.6 ^b^	6.0 ^a^				
NH_3_-N, mg/100 mL	CON	10.6 ^bc^	15.7 ^ab^	17.5 ^ab^	1.048	0.999	<0.001	0.040
	EW	8.1 ^c^	12.9 ^bc^	22.8 ^a^				
Total VFA, mmol/L	CON	66.4 ^b^	106.5 ^ab^	92.9 ^ab^	4.884	0.027	0.049	0.197
	EW	109.7 ^ab^	122.0 ^a^	97.6 ^ab^				
Molar proportion of VFA, mol/100 mol								
Acetate	CON	50.9	50.1	52.8	1.234	0.804	0.676	0.340
	EW	49.3	55.8	50.2				
Propionate	CON	24.1	26.4	25.0	0.775	0.154	0.744	0.482
	EW	29.4	27.4	26.2				
Butyrate	CON	20.1	16.5	16.7	1.331	0.171	0.379	0.526
	EW	14.8	10.0	17.1				
Valerate	CON	1.9 ^b^	5.3 ^a^	3.1 ^ab^	0.378	0.098	0.015	0.185
	EW	4.9 ^ab^	5.6 ^a^	3.5 ^ab^				
Isobutyrate	CON	1.0 ^ab^	0.8 ^ab^	0.9 ^ab^	0.075	0.991	0.033	0.068
	EW	0.8 ^ab^	0.6 ^b^	1.3 ^a^				
Isovalerate	CON	1.9 ^a^	0.9 ^ab^	1.5 ^ab^	0.135	0.159	0.01	0.041
	EW	0.7 ^b^	0.7 ^b^	1.8 ^a^				
Acetate/Propionate ratio	CON	2.1	2.1	2.3	0.099	0.253	0.71	0.616
	EW	1.7	2.1	1.9				

^1^ Lambs weaned at day 21 as early weaned group (EW) or lambs weaned at day 49 as conventional weaned group (CON). Values are presented as means; n = 6 per group. ^2^ Treatment = the effect of weaning time; age = the effect of age; treatment × age = the interaction between treatment and age. SEM = standard error of the mean. ^a–c^ Means within two rows (six values including age and treatment) with different superscripts differ (*p* < 0.05).

**Table 4:** Serum parameters of lambs at 26, 35, and 63 days of age ^1.^

Item ^2^	Treatment	Day of Age			SEM ^3^	*p*-Value ^3^		
		26	35	63		Treatment	Age	Treatment × Age
IgG, g/L	CON	24.0 ^a^	22.5 ^a^	19.0 ^b^	0.422	0.761	<0.001	0.454
	EW	22.8 ^a^	22.8 ^a^	19.4 ^b^				
IgA, g/L	CON	0.5 ^c^	0.6 ^bc^	0.7 ^a^	0.014	0.809	<0.001	0.658
	EW	0.6 ^c^	0.6 ^c^	0.7 ^ab^				
IgM, g/L	CON	1.1 ^b^	1.1 ^b^	1.3 ^a^	0.026	0.889	<0.001	0.846
	EW	1.1 ^b^	1. 1^b^	1.3 ^a^				
SOD, U/mL	CON	93.5 ^b^	111.5 ^a^	94.8 ^b^	1.548	0.839	<0.001	0.556
	EW	96.7 ^b^	110.4 ^a^	93.8 ^b^				
GSH-Px, µmol/L	CON	754.7 ^bc^	818.6 ^a^	732.4 ^c^	8.645	0.290	<0.001	0.199
	EW	712.7 ^c^	811.1 ^ab^	743.4 ^c^				
CAT, U/mL	CON	7.6 ^c^	8.9 ^a^	7.8 ^bc^	0.118	0.145	<0.001	0.844
	EW	7.5 ^c^	8.6 ^ab^	7.6 ^c^				
T-AOC, U/mL	CON	8.4 ^c^	9.8 ^a^	8.8 ^bc^	0.115	0.605	<0.001	0.134
	EW	8.9 ^bc^	9.5 ^ab^	8.9 ^bc^				
MDA, nmol/mL	CON	5.3 ^a^	4.3 ^b^	5.3 ^a^	0.112	0.464	<0.001	0.948
	EW	5.5 ^a^	4.4 ^b^	5.4 ^a^				
Cortisol, µg/dL	CON	1.5 ^b^	2.7 ^a^	1.6 ^b^	0.107	0.946	<0.001	0.695
	EW	1.6 ^b^	2.6 ^a^	1.5 ^b^				
IL-1β, pg/mL	CON	98.9 ^b^	111.4 ^a^	99.7 ^b^	1.534	0.659	<0.001	0.471
	EW	97.1 ^b^	114.0 ^a^	96.1 ^b^				
IL-4, pg/mL	CON	13.3 ^c^	14.3 ^ab^	13.2 ^c^	0.124	0.361	<0.001	0.850
	EW	13.4 ^bc^	14.6 ^a^	13.3 ^c^				
IL-6, pg/mL	CON	45.9 ^c^	54.2 ^ab^	45.8 ^c^	1.055	0.085	<0.001	0.356
	EW	47.5 ^bc^	59.2 ^a^	46.2 ^c^				
IFN-γ, pg/mL	CON	143.2 ^b^	159.0 ^a^	141.9 ^b^	1.443	0.843	<0.001	0.772
	EW	144.2 ^b^	157.1 ^a^	141.9 ^b^				
TNF-α, pg/mL	CON	54.4 ^b^	67.5 ^a^	55.7 ^b^	1.133	0.927	<0.001	0.549
	EW	56.4 ^b^	66.3 ^a^	54.5 ^b^				

^1^ Values are presented as means; n = 6 per group. Lambs weaned at day 21 as early weaned group (EW) or lambs weaned at day 49 as conventional weaned group (CON). ^2^ IgG = immunoglobulin G, IgM = immunoglobulin M, IgA = immunoglobulin A, SOD = superoxide dismutase, GSH-Px = Glutathione Peroxidase, CAT = catalase, T-AOC = total antioxidative capacity, MDA = malondialdehyde IL-1β = interleukin 1β, IL-4 = interleukin 4, IL-6 = interleukin 6, IFN-γ = interferon-gamma, TNF-α = tumor necrosis factor α. ^3^ Treatment = the effect of weaning time; age = the effect of age; treatment × age = the interaction between treatment and age. SEM = standard error of the mean. ^a–c^ Means within two rows (six values including age and treatment) with different superscripts differ (*p* < 0.05).
